# Prophylactic Supplementation with *Lactobacillus Reuteri* or Its Metabolite GABA Protects Against Acute Ischemic Cardiac Injury

**DOI:** 10.1002/advs.202307233

**Published:** 2024-03-15

**Authors:** Jiawan Wang, Hao Zhang, Hailong Yuan, Siqi Chen, Ying Yu, Xuan Zhang, Zeyu Gao, Heng Du, Weitao Li, Yaohui Wang, Pengyan Xia, Jun Wang, Moshi Song

**Affiliations:** ^1^ State Key Laboratory of Membrane Biology Institute of Zoology Chinese Academy of Sciences Beijing 100101 China; ^2^ Beijing Institute for Stem Cell and Regenerative Medicine Beijing 100101 China; ^3^ Key Laboratory of Organ Regeneration and Reconstruction Chinese Academy of Sciences Beijing 100101 China; ^4^ Beijing Chao‐Yang Hospital Department of Anesthesiology Beijing 100020 China; ^5^ University of Chinese Academy of Sciences Beijing 100049 China; ^6^ Joint National Laboratory for Antibody Drug Engineering Henan University Kaifeng 475004 China; ^7^ CAS Key Laboratory of Pathogenic Microbiology and Immunology Chinese Academy of Sciences Beijing 100101 China; ^8^ Department of Immunology School of Basic Medical Sciences Peking University Beijing 100191 China

**Keywords:** acute ischemic cardiac injury, GABA, *Lactobacillus reuteri*, microbiota‐derived metabolite, prophylactic supplementation

## Abstract

The gut microbiome has emerged as a potential target for the treatment of cardiovascular disease. Ischemia/reperfusion (I/R) after myocardial infarction is a serious complication and whether certain gut bacteria can serve as a treatment option remains unclear. *Lactobacillus reuteri* (*L. reuteri*) is a well‐studied probiotic that can colonize mammals including humans with known cholesterol‐lowering properties and anti‐inflammatory effects. Here, the prophylactic cardioprotective effects of *L. reuteri* or its metabolite γ‐aminobutyric acid (GABA) against acute ischemic cardiac injury caused by I/R surgery are demonstrated. The prophylactic gavage of *L. reuteri* or GABA confers cardioprotection mainly by suppressing cardiac inflammation upon I/R. Mechanistically, GABA gavage results in a decreased number of proinflammatory macrophages in I/R hearts and GABA gavage no longer confers any cardioprotection in I/R hearts upon the clearance of macrophages. In vitro studies with LPS‐stimulated bone marrow‐derived macrophages (BMDM) further reveal that GABA inhibits the polarization of macrophages toward the proinflammatory M1 phenotype by inhibiting lysosomal leakage and NLRP3 inflammasome activation. Together, this study demonstrates that the prophylactic oral administration of *L. reuteri* or its metabolite GABA attenuates macrophage‐mediated cardiac inflammation and therefore alleviates cardiac dysfunction after I/R, thus providing a new prophylactic strategy to mitigate acute ischemic cardiac injury.

## Graphical abstract

The gut microbiome has emerged as a potential target for the treatment of cardiovascular disease. It is found that the prophylactic oral administration of *Lactobacillus reuteri* or its metabolite GABA attenuates macrophage‐mediated cardiac inflammation and therefore alleviates cardiac dysfunction after myocardial ischemia reperfusion, thus providing a new prophylactic strategy to mitigate acute ischemic cardiac injury.

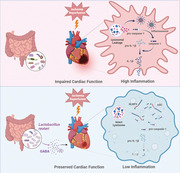

## 1 Introduction

Coronary heart disease (CHD) accounts for nearly one‐third of deaths worldwide, despite the increasing use of medication such as statin therapy in the last decades. Among them, acute myocardial infarction (MI) is the most fatal, with three million cases worldwide occurring annually.^[^ ^1^ ^]^ Thus, developing more effective strategies for the prevention and treatment of acute MI is highly desired. Currently, the most commonly used primary percutaneous coronary intervention results in reduced mortality and morbidity after acute MI, compared with thrombolytic therapy. However, ischemia/reperfusion (I/R) injury remains a serious complication, contributing to up to 50% of the final infarct size. I/R injury is a combined process containing metabolic disorders, inflammation, oxidative stress, and microvascular obstruction, and severely affects the prognosis and fatality of MI patients. Thus, developing effective treatment or preventive measures is of great value to medicine and clinical practice.

Multiple studies have linked alterations of gut microbiome with a variety of cardiovascular diseases including hypertension, heart failure, and ischemic heart diseases such as myocardial infarction.^[^ ^2^, ^3^ ^]^ The influence of the microbiome on the cardiovascular system is mainly associated with their diverse metabolites.^[^ ^4^, ^5^ ^]^ One example is trimethylamine (TMA) which originates from the bacterial conversion of choline, phosphatidylcholine, or l‐carnitine, which has been identified as a major driver of cardiovascular risks in early research.^[^ ^6^ ^]^ It contributes to cardiovascular diseases by affecting foam cells and endothelial cells, vascular inflammation, atherosclerotic lesions, and fibrosis, and by enhancing platelet aggregation and thrombosis after being oxidized to trimethylamine N‐oxide (TMAO) in the liver.^[^ ^7^ ^]^ Another category of microbiota‐derived metabolites associated with cardiovascular diseases is short‐chain fatty acids (SCFA), shown to alleviate cardiovascular diseases via the modulation of both metabolic dysbiosis and reduction of inflammation. Reduced abundances of SCFA producers, such as *Eubacterium rectale*, *Dorea longicatena*, *Clostridium clostridioforme*, and *F. prausnitzii*, are associated with heart failure^[^ ^8^ ^]^ and atherosclerosis.^[^ ^9^ ^]^ With respect to MI, it has been shown that the absence of *P. copri* in the gut was associated with an 18% lower risk of MI under Mediterranean diet in humans.^[^ ^10^ ^]^

With the increasing recognition of the gut microbiome's role in the pathogenesis of cardiovascular diseases, the potential of modulating the gut microbiome for protective and/or therapeutic usage has also attracted attention. First‐line drugs against diabetes (metformin) and hyperlipidemia (statins) are known to modulate the structure and metabolome of the gut microbiome,^[^ ^11^, ^12^ ^]^ and such changes contribute to host glucose and lipid metabolism to reduce the incidences of MI.^[^ ^13^, ^14^ ^]^ Administration of probiotics has been tested in rat models of MI, using strains such as *Lactobacillus rhamnosus* GR‐1,^[15^ ^]^ *Lactobacillus plantarum*, or *Bifidobacterium lactis* ,^[16^ ^]^ and the results showed positive outcomes such as reduction in infarct size and improvement in myocardial function. Yet, the key metabolites responsible for their protective effects in MI have not been determined, preventing a better understanding of the exact mechanism or process of action. Moreover, there have not been studies reporting the usage of probiotics with respect to I/R injury, the complication that largely affects the outcome after MI.

We thus examined *Lactobacillus reuteri* (*L. reuteri*) which has well‐documented effects as a probiotic, and additionally, it has been reported to be positively associated with the level of anti‐inflammatory cytokine interleukin‐10 (IL‐10)^[^ ^17^ ^]^ and thus has the potential to alleviate the inflammation‐dominant process of I/R. Our results demonstrate that *L. reuteri* confers cardioprotection against I/R, which can be recapitulated by one important metabolite γ‐aminobutyric acid (GABA) that reduces NLRP3 inflammasome activation in macrophages and the consequent inflammation.

## 2 Results

### 2.1 Prophylactic Gavage with *L. Reuteri* Attenuated Cardiac Dysfunction and Inflammation After I/R

**Figure 1:**
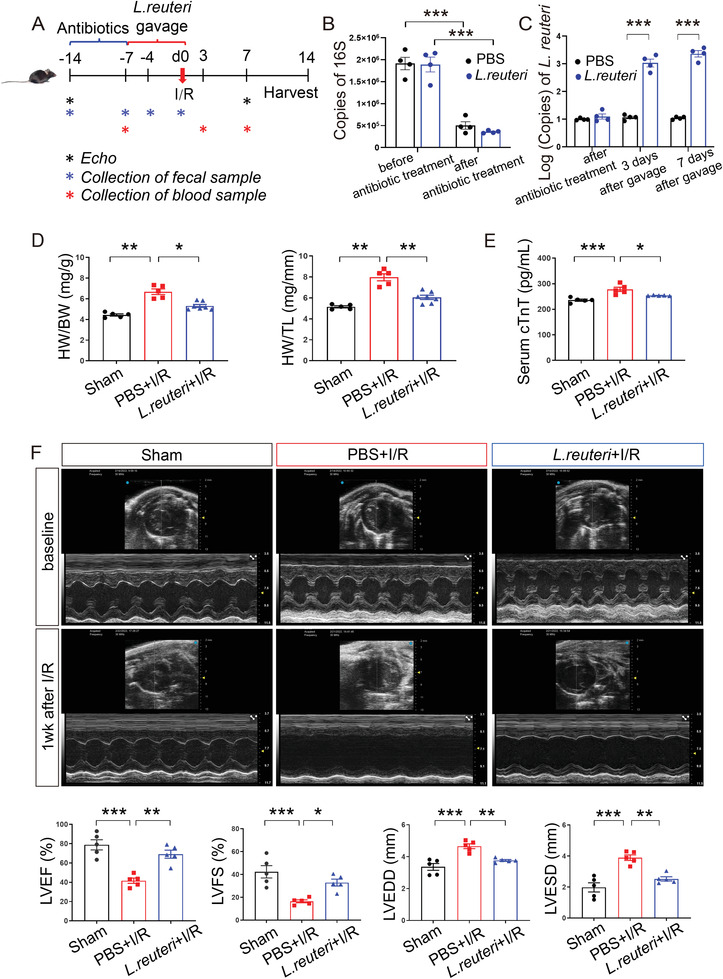
Pretreatment of *L. reuteri* ameliorated cardiac damage after I/R. A) Schematics of the experiment with *L. reuteri* gavage. Male C57BL/6J mice were pretreated with 7 days of antibiotics to remove gut microbiota and 7 days of *L. reuteri* gavage before I/R. Serum samples were collected at 7 days before I/R, 3 days, and 7 days after I/R. Fecal samples were collected before and after antibiotic treatment, 3 days and 7 days after *L. reuteri* gavage. Cardiac function was detected by transthoracic echocardiography (Echo) at baseline and 7 days after I/R. Heart tissues were harvested at 14 days after I/R for histological assessment. B) The copies of bacterial 16S detected in the feces collected before and after antibiotic treatment. Data are shown as the mean ± SEMs, *n* = 4 per group. ^***^, *p* < 0.001 (Student's *t*‐test). C) The copies of *L. reuteri* detected in the feces collected after antibiotic treatment and at 3 days and 7 days after *L. reuteri* gavage. Data are shown as the mean ± SEMs, *n* = 4 per group. ^***^, *p* < 0.001 (Student's *t*‐test). D) Ratio of heart weight to body weight (HW/BW) and heart weight to tibial length (HW/TL) at 2 weeks after I/R. Data are shown as the mean ± SEMs. Sham group (Sham), *n* = 5; PBS‐gavaged I/R group (PBS+I/R), *n* = 5; *L. reuteri*‐gavaged I/R group (*L. reuteri*+I/R), *n* = 7. ^*^, *p* < 0.05; ^**^, *p* < 0.01 (one‐way ANOVA with post hoc Dunnett's test). E) Serum cTnT levels measured by ELISA at 3 days after I/R. *n* = 5 per group. ^*^, *p* < 0.05; ^***^, *p* < 0.001 (one‐way ANOVA with post hoc Tukey test). (F) Representative echocardiographic images at baseline and 1 week after I/R. Quantitative data on left ventricular ejection fraction (LVEF), fractional shortening (LVFS), left ventricular end‐diastolic dimension (LVEDD), and left ventricular end‐systolic dimension (LVESD) at seven days after I/R are shown as the mean ± SEMs; *n* = 5 per group. ^*^, *p* < 0.05; ^**^, *p* < 0.01; ^***^, *p* < 0.001 (one‐way ANOVA with post hoc Tukey test). *L. reuteri, Lactobacillus reuteri*; I/R, ischemia/reperfusion. cTnT, cardiac troponin T.

Previous studies have reported the anti‐inflammatory effects of *L. reuteri* ^[^ ^18^ ^]^ and found the gavage of this bacteria reduced cardiac injury in low‐density lipoprotein receptor (LDLR)‐deficient mice^[^ ^19^ ^]^ we set out to explore the cardioprotective potential of *L. reuteri* in the context of I/R (**Figure** **1**A). Male C57BL/6J were given oral antibiotics for seven days to deplete the original gut microbiota to facilitate the colonization of *L. reuteri* (copies of bacterial 16S, *p* < 0.001, Figure 1B) and daily gavage of *L. reuteri* as a prophylactic (2 × 10^8^ CFU per mouse) or PBS (control) was carried out daily for 7 days. The colonization of the *L. reuteri* was confirmed using 16S rRNA quantification after gavage (copies of *L. reuteri*, *p* < 0.001 at three and seven days after gavage, Figure 1C). We then performed left anterior descending (LAD) coronary artery ligation to induce the I/R model; to exclude potential confounding effects from the operation, an additional sham group was subjected to a similar operational procedure, excepting ligation of the coronary artery (Figure 1A). Successful model induction was supported by induced heart failure, as the PBS‐gavaged I/R group showed significantly increased heart‐to‐body weight ratio (*p* < 0.01) and heart‐to‐tibia length ratio (*p* < 0.01) at two weeks after the surgery (Figure 1D). Compared to the PBS‐gavaged I/R group, *L. reuteri*‐gavaged I/R group had significantly reduced heart‐to‐body weight ratio (*p* < 0.05) and heart‐to‐tibia length ratio (*p* < 0.01) (Figure 1D). In addition, serum cardiac troponin T (cTnT), a marker for heart tissue damage, was significantly lowered in *L. reuteri*‐gavaged I/R group as compared to the PBS‐gavaged I/R group (3 days, *p* < 0.05; 1 week, *p* < 0.05) (Figure 1E; Figure S1A, Supporting Information).

We then evaluated the effects on cardiac function by the prophylactic *L. reuteri* gavage on I/R models, using thoracic echocardiology that monitored cardiac contractility and left ventricular (LV) chamber size at one week after I/R (Figure 1F). We observed a significantly decreased cardiac contractility and a significantly increased left ventricular (LV) chamber size in the PBS‐gavaged I/R group compared to those of the Sham group (Figure 1F). Compared to the PBS‐gavaged I/R group, *L. reuteri*‐gavaged I/R group had significantly higher percentages of left ventricular ejection fraction (LVEF%, *p* < 0.01) and fractional shortening (LVFS%, *p* < 0.05), along with significantly lower left ventricular end‐diastolic dimension (LVEDD, *p* < 0.01) and left ventricular end‐systolic dimension (LVESD, *p* < 0.01) (Figure 1E), indicating an attenuation of cardiac injury after I/R by the prophylactic *L. reuteri* gavage. Additionally, electrocardiogram (ECG) analysis revealed an attenuation of ST‐segment elevation in *L. reuteri*‐gavaged I/R group (Figure S1B, Supporting Information). To summarize, our results point to a cardioprotective effect via the reduction of I/R‐induced cardiac injury by the prophylactic *L. reuteri* gavage.

**Figure 2:**
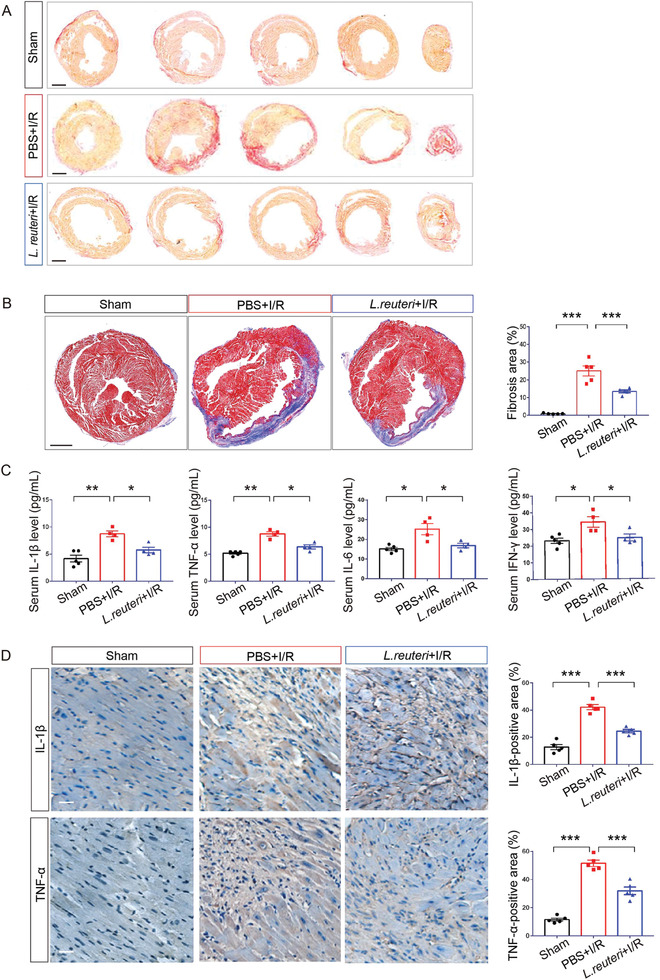
Pretreatment of *L. reuteri* alleviated cardiac fibrosis and inflammation after I/R. A) Representative images of transverse cardiac sections stained with picrosirius red at 2 weeks after I/R. Scale bar, 2 mm. B) Representative images of Masson's trichrome staining of heart tissues. Red denotes viable cardiomyocytes and blue denotes fibrosis at 2 weeks after I/R. Scale bar, 2 mm. Quantification of fibrotic area are shown as the mean ± SEMs; *n* = 5 per group. ^***^, *p* < 0.001 (one‐way ANOVA with post hoc Tukey test). C) Serum inflammatory cytokines measured by ELISA at 1 week after I/R. Sham, *n* = 5; PBS+I/R, *n* = 4; *L. reuteri* +I/R, *n* = 4. ^*^, *p* < 0.05; ^**^, *p* < 0.01 (one‐way ANOVA with post hoc Dunnett's test). D) High‐magnification images of immunohistochemistry staining for IL‐1β and TNF‐α at 2 weeks after I/R. Brown color indicates the cells with positive expression. Scale bar, 50 µm. Quantitative data are shown to the right as the mean ± SEMs, *n* = 5 per group. ^***^, *p* < 0.001 (one‐way ANOVA with post hoc Tukey test).

We then examined heart tissues with Picrosirius red staining and Masson's trichrome staining and found significantly reduced cardiac injury and fibrosis in the hearts of *L. reuteri*‐gavaged I/R group (*p* < 0.001) at two weeks after the surgery (**Figure** **2**A,B). Previous studies have demonstrated the central role of inflammation in I/R, particularly that inflammation exacerbates cardiac injury.^[^ ^20^, ^21^ ^]^ Accordingly, we measured the serum levels of proinflammatory markers to determine the levels of systemic inflammation in *L. reuteri‐*gavaged I/R group versus the PBS‐gavaged I/R group. We found that multiple proinflammatory cytokines including interleukin‐1β (IL‐1β), tumor necrosis factor‐α (TNF‐α), interleukin‐6 (IL‐6), and interferon‐γ (IFN‐γ) increased after I/R and that the prophylactic gavage of *L*. *reuteri* resulted in significantly suppressed the I/R‐induced cytokine production (IL‐1β, *p* < 0.05; TNF‐α, *p* < 0.05; IL‐6, *p* < 0.05; and IFN‐γ, *p* < 0.05, compared to PBS‐gavaged I/R group) (Figure 2C). Additionally, in the heart tissues, we quantified the levels of IL‐1β and TNF‐α by immunohistochemistry and revealed that the levels of both proinflammatory cytokines increased after I/R (*p* < 0.001), and were suppressed by the prophylactic *L. reuteri* gavage (*p* < 0.001, compared to PBS‐gavaged I/R group) (Figure 2D). Collectively, these results suggest that the prophylactic *L*. *reuteri* gavage reduces the levels of systemic and local inflammations after I/R.

### 2.2 GABA Recapitulated the Prophylactic Effect of *L*. *Reuteri* in I/R

**Figure 3:**
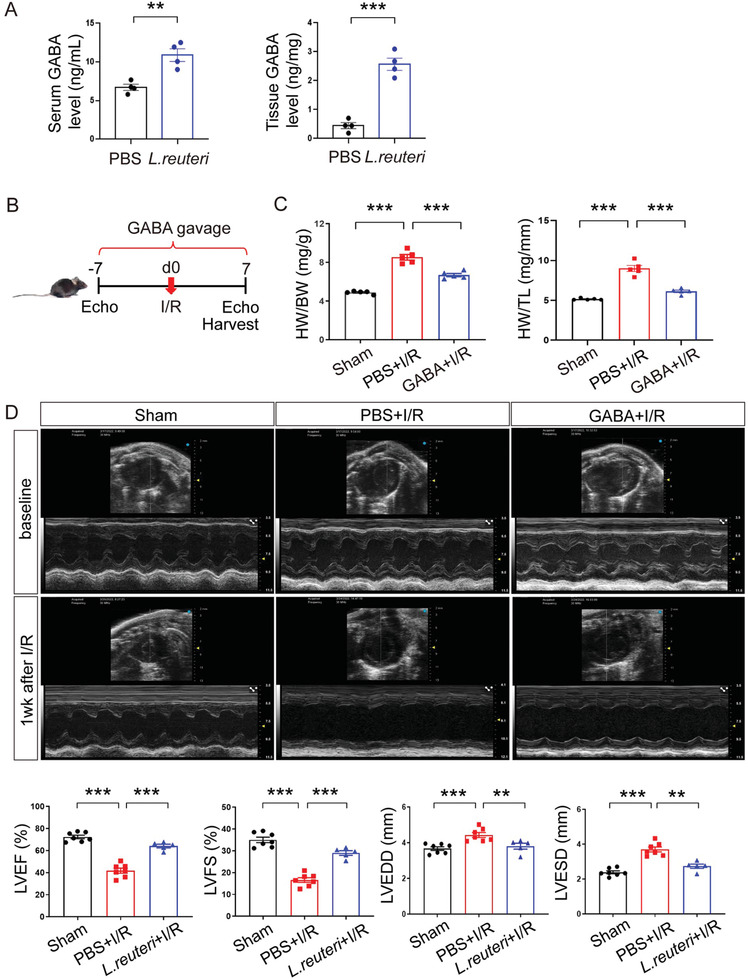
GABA recapitulated the prophylactic effect of *L. reuteri* against I/R. A) Levels of GABA detected in the serum and heart tissues by ELISA after 7 days of continuous GABA gavage. Data are shown as the mean ± SEMs. *n* = 4 per group. ^**^, *p* < 0.01; ^***^, *p* < 0.001 (Student's *t*‐test). B) Scheme of the experiment with GABA gavage. Male C57BL/6J mice were treated with GABA gavage for 7 days before I/R and then continued for another 7 days after I/R. Cardiac function was detected by transthoracic echocardiography at baseline and 7 days after I/R. C) The ratios of heart weight to body weight (HW/BW) and heart weight to tibial length (HW/TL) at 7 days after I/R. Data are shown as the mean ± SEMs. *n* = 5 per group. ^***^, *p* < 0.001 (one‐way ANOVA with post hoc Tukey test). D) Representative echocardiographic images at baseline and 7 days after I/R. Quantitative data on LVEF, LVFS, LVEDD, and LVESD at 7 days after I/R are shown as the mean ± SEMs. Sham group (Sham), *n* = 7; PBS‐gavaged I/R group (PBS+I/R), *n* = 7; GABA‐gavaged I/R group (GABA+I/R), *n* = 5. ^**^, *p* < 0.01; ^***^, *p* < 0.001 (one‐way ANOVA with Dunnett's test).

As the cardioprotective effects of *L*. *reuteri* can be carried out with oral gavage, we postulate that the small molecule metabolites of this bacteria might be responsible. Previous studies have demonstrated extensive GABA production and secretion by *L*. *reuteri*,^[^ ^22^, ^23^ ^]^ we therefore measured GABA levels in PBS‐gavaged I/R and *L. reuteri*‐gavaged I/R groups and found that GABA levels increased significantly in both serum and heart tissue of *L. reuteri*‐gavaged mice compared to those gavaged with PBS (serum GABA level, *p* < 0.01 and GABA level in heart tissue, *p* < 0.001) (**Figure** **3**A).

Next, we investigated the effects of the prophylactic gavage of GABA in I/R mice at seven days after the surgery and found that, without the antibiotic removal of the original gut microbiome, GABA gavage (60 mg kg^−1^ day^−1^) recapitulated the cardioprotective effects of the prophylactic *L. reuteri* gavage after I/R (Figure 3B). Specifically, GABA gavage significantly suppressed I/R‐induced increases in heart‐to‐body weight ratio (*p* < 0.001) and heart‐to‐tibia length ratio (*p* < 0.001) (Figure 3C). In addition, GABA gavage mitigated cardiac dysfunction induced by I/R, as evidenced by significantly higher percentages of LVEF (*p* < 0.001) and LVFS (*p* < 0.001), along with significantly lower LVEDD (*p* < 0.01) and LVESD (*p* < 0.01) (Figure 3D).

**Figure 4:**
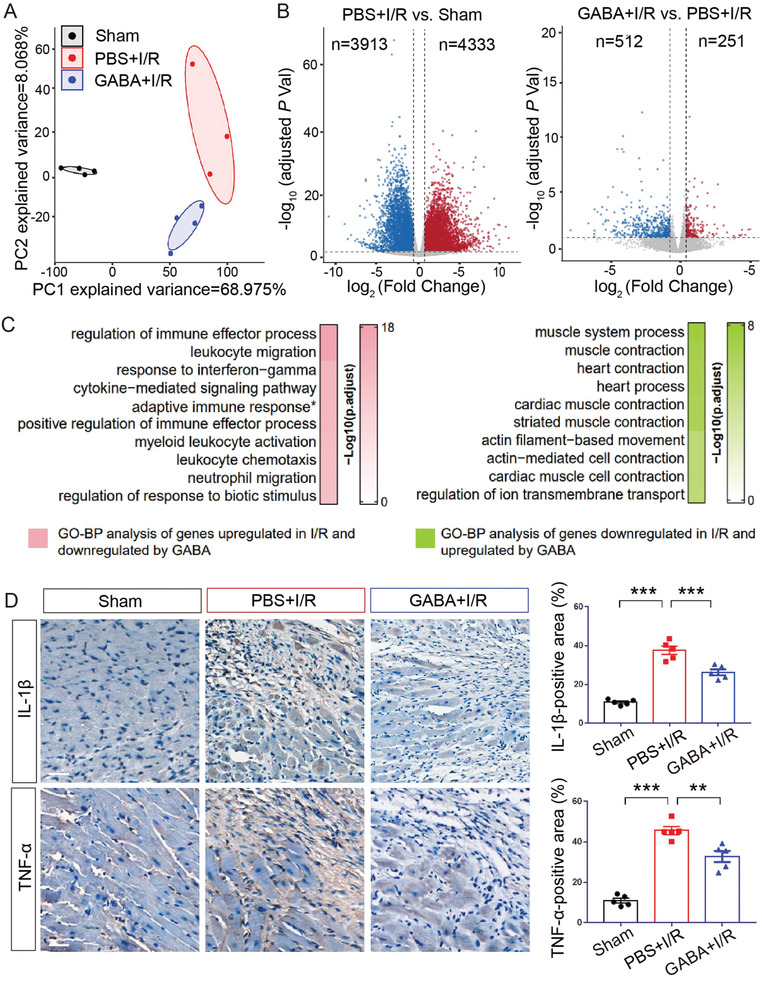
GABA downregulated the proinflammatory genes after I/R. A) Principal component analysis (PCA) using all the identified genes from RNA sequencing data for Sham (black), PBS+I/R (red), and GABA+I/R (blue) reveals in‐group clusters with minimal overlap. B) Volcano plots illustrating differentially expressed genes identified in PBS+I/R versus Sham groups (left panel) and in GABA+I/R versus PBS+I/R groups (right panel). The blue dots denote downregulated genes, the red dots denote upregulated genes and the grey dots denote genes without significantly differential expression. C) Top 10 enriched terms of the genes by Gene Ontology biological processes (GO‐BP). Left panel, top 10 terms of the genes that were upregulated in PBS+I/R versus Sham and downregulated in GABA+I/R versus PBS+I/R (pink). Right panel, top 10 terms of the genes that were downregulated in PBS+I/R versus Sham and upregulated in GABA+I/R versus PBS+I/R (green). D) High‐magnification images of immunohistochemistry staining for IL‐1β and TNF‐α at seven days after I/R. Brown color indicates the cells with positive expression. Scale bar, 50 µm. Quantitative data are shown to the right as the mean ± SEMs, *n* = 5 per group. ^**^, *p* < 0.01; ^***^, *p* < 0.001 (one‐way ANOVA with post hoc Tukey test). GABA, γ‐aminobutyric acid; adaptive immune response^*^, short for “adaptive immune response based on somatic recombination of immune receptors built from immunoglobulin superfamily domains”.

To gain insights into the mechanism of GABA‐conferred cardioprotection against I/R injury, we collected the infarcted heart tissues from the PBS‐gavaged I/R group and GABA‐gavaged I/R group as well as the same area from Sham group at seven days after the surgery and performed RNA‐seq analysis (**Figure** 4A). Compared to Sham group, 4,333 upregulated genes, and 3,913 downregulated genes were identified in the PBS‐gavaged I/R group (Figure 4B), suggesting global transcriptional changes induced by I/R. Notably, 411 I/R‐upregulated genes and 164 I/R‐downregulated genes were reversed by the prophylactic GABA gavage (Figure S2A,B, Supporting Information). Further, we performed gene ontology (GO) enrichment analysis on the GABA‐rescued genes using gene ontology biological processes (GO‐BP). GABA‐upregulated pathways were primarily associated with heart contraction and heart process (nine among the top 10 enriched pathways) (Figure 4C), including cardiac muscle contraction, actin filament‐based movement, and muscle system process, indicating improved cardiac function by GABA. GABA‐downregulated pathways were mainly associated with inflammatory response (eight among the top 10 enriched pathways) (Figure 4C), including regulation of immune effector process, activation of immune response, and cytokine‐mediated signaling pathway, pointing to an anti‐inflammatory effect of GABA. Indeed, when we quantified the levels of IL‐1β and TNF‐α by immunohistochemistry in the heart tissues at seven days after the surgery, we found that the levels of both proinflammatory cytokines increased after I/R (IL‐1β, *p* < 0.001; TNF‐α, *p* < 0.001), and were suppressed by GABA gavage (IL‐1β, *p* < 0.001 and TNF‐α, *p* < 0.01, compared to PBS‐gavaged I/R group) (Figure 4D).

### 2.3 GABA Mitigated Cardiac Injury by Reducing Proinflammatory Macrophages

**Figure 5:**
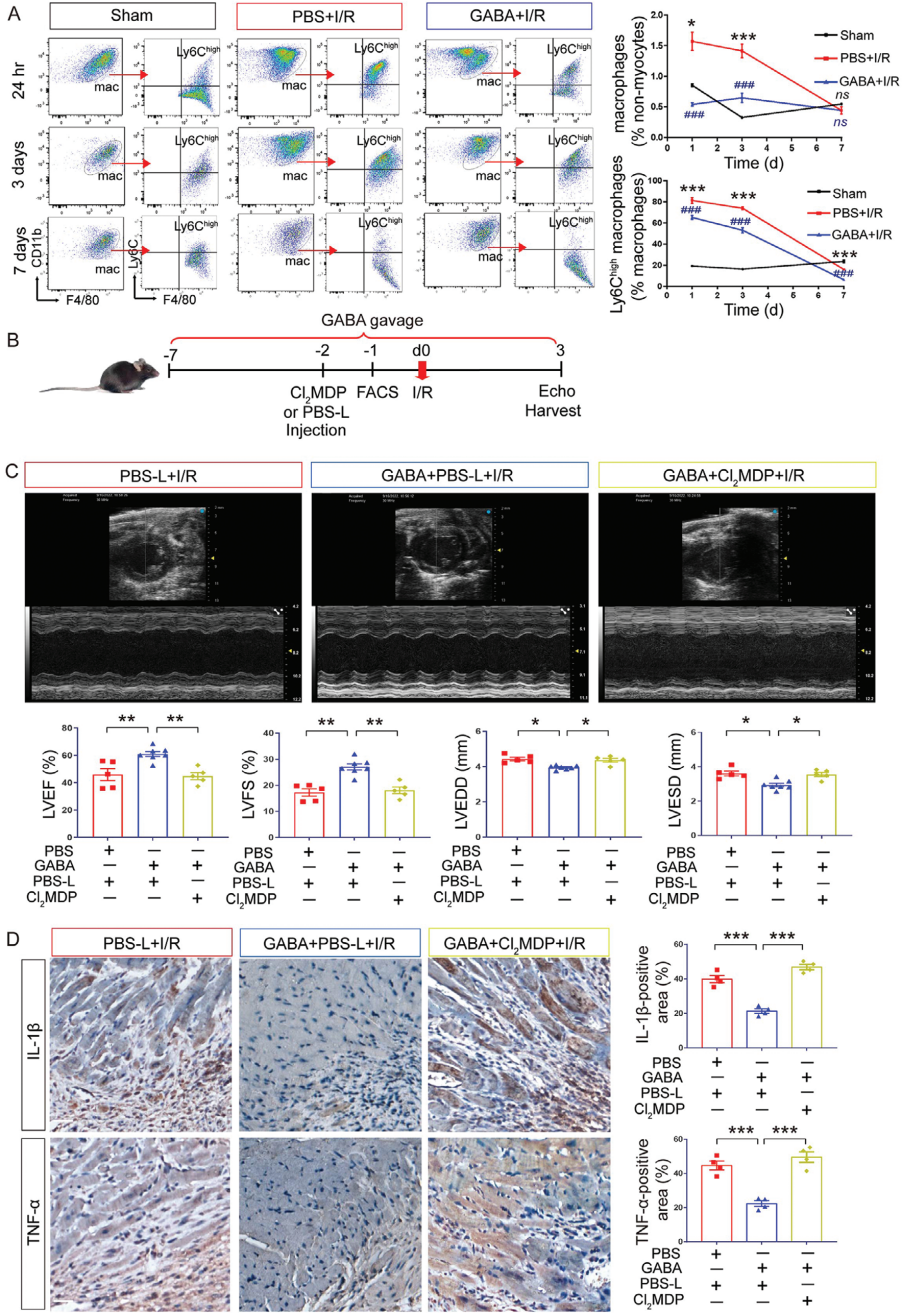
GABA reduced the level of cardiac inflammation after I/R mainly by reducing proinflammatory macrophages. A) Representative flow cytometric dot plots of macrophages and Ly6C^high^ macrophages from Sham, PBS+I/R, and GABA+I/R groups at 24 h, 3 days and 7 days after I/R (left panel). Flow cytometry‐based quantification of F4/80^+^CD11b^+^ macrophages in non‐myocytes and Ly6C^high^ macrophages in all the macrophages from Sham, PBS+I/R and GABA+I/R groups at 24 h, 3 days and 7 days after I/R (right panel). *ns*, not significant; ^*^, *p* < 0.05; ^***^, *p* < 0.001 (PBS+I/R vs Sham, one‐way ANOVA with post hoc Tukey test); ^###^, *p* < 0.001 (GABA+I/R vs PBS+I/R, one‐way ANOVA with post hoc Tukey test). Data are presented as mean ± SEM, *n* = 4 per group at each time point. B) Schematics of the experiment in mice with macrophage depletion. Clodronate liposomes (Cl_2_MDP) were intravenously injected at 2 days before I/R and the resultant macrophage depletion in vivo was confirmed by flow cytometry‐based analysis at 1 day before I/R. Mice injected with PBS liposomes (PBS‐L) were used as control. Cardiac function was assessed by transthoracic echocardiography on day 3 after I/R with or without the pretreatment of GABA. C) Representative echocardiographic images on day 3 after I/R (upper panel). Quantitative data on LVEF, LVFS, LVEDD, and LVESD were shown as the mean ± SEMs (lower panel). PBS‐L+I/R (red bar), *n* = 5; GABA+PBS‐L+I/R (blue bar), *n* = 7; GABA+Cl_2_MDP+I/R (yellow bar), *n* = 5. ^*^, *p* < 0.05; ^**^, *p* < 0.01 (one‐way ANOVA with post hoc Dunnett's test). D) High‐magnification images of immunohistochemistry staining for IL‐1β and TNF‐α in the border area. Brown color indicates the cells with positive expression. Scale bar, 50 µm. Quantitative data are shown to the right as the mean ± SEMs, *n* = 4 per group. ^***^, *p* < 0.001 (one‐way ANOVA with post hoc Tukey test). mac, macrophage; GABA, γ‐aminobutyric acid.

After establishing that the prophylactic GABA gavage mitigates cardiac injury after I/R and reduces associated inflammation as well, we examined the immune cell population(s) affected by GABA. We performed flow cytometry analysis of single‐cell suspensions prepared from mouse hearts after I/R examining CD45^+^ immune cells including macrophages, neutrophils, B cells, and T cells (**Figure** **5**A; Figure S3A–C, Supporting Information), and found that GABA gavage led to a significant reduction only in macrophages at the acute inflammatory phase at both 24 h and three days after I/R compared to PBS‐gavaged I/R group (24 h, *p* < 0.001; 3 days, *p* < 0.001) (Figure 5A). By comparison, no difference in the number of macrophages was observed in the presence or absence of GABA at the proliferative healing phase at seven days after I/R (*p* = 0.8771) (Figure 5A). Previous studies have suggested that macrophages play an integral role in the initial inflammatory response to injury in many tissues and organs, and in myocardial infarction, the circulating monocytes usually differentiate into Ly6C^high^ macrophages (also known as M1 proinflammatory macrophages) in the acute inflammatory phase.^[^ ^24^ ^]^ We thus performed a continual quantification of the proinflammatory cardiac Ly‐6C^high^ macrophages at 24 h, three days, and seven days after I/R, which showed that GABA significantly reduced the Ly‐6C^high^ monocyte infiltration in the infarcted myocardium compared to PBS‐gavaged I/R group in both acute inflammatory and proliferative healing phases (24 h, *p* < 0.001; Day 3, *p* < 0.001; Day 7, *p* < 0.001) (Figure 5A).

To confirm that GABA's cardioprotective effect against I/R injury by suppressing inflammation mainly relied on macrophages, we performed in vivo macrophage clearance using clodronate liposomes^[^ ^25^, ^26^ ^]^ to deplete macrophages. Upon the confirmation of macrophage depletion by FACS analysis one day after the injection of clodronate liposomes (Figure S3D, Supporting Information), mice were subjected to I/R surgery and downstream analyses (Figure 5B). The results showed that GABA gavage failed to confer cardioprotection in I/R hearts after macrophage clearance (Figure 5C). Immunohistochemistry analysis revealed that GABA gavage failed to suppress the levels of proinflammatory cytokines TNF‐α and IL‐1β in I/R hearts after macrophage clearance (Figure 5D). Thus, we conclude that the GABA's effect in reducing cardiac injury and inflammation is dependent on macrophages. In short, these data suggest that GABA gavage reduces the number of proinflammatory M1 macrophages, thus suppressing inflammation and reducing cardiac injury after I/R.

### 2.4 GABA Inhibited M1 Macrophage Polarization by Suppressing the Activation of NLRP3 Inflammasome

**Figure 6:**
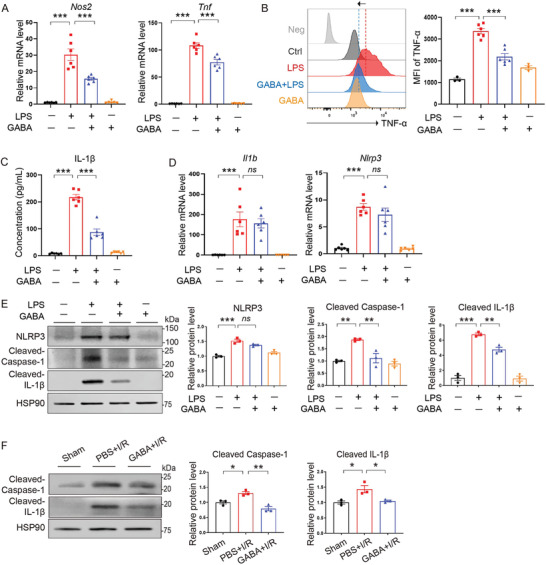
GABA inhibited the polarization of macrophages toward proinflammation. A) qRT‐PCR analysis showing the mRNA levels of *Nos2* and *Tnf* in LPS‐stimulated BMDM in the presence or absence of GABA. *n* = 6 per group. ^***^, *p* < 0.001 (one‐way ANOVA with post hoc Tukey test). B) Flow cytometry analysis showing the mean fluorescence intensity (MFI) of M1 surface marker TNF‐α in macrophages. *n* = 3 in Ctrl and GABA group, *n* = 6 in LPS and LPS+GABA group. ^***^, *p* < 0.001 (one‐way ANOVA with post hoc Dunnett's test). C) Measurement of IL‐1β secretion in cell culture supernatants by ELISA. Data are shown as the mean ± SEMs, *n* = 6 per group; ^***^, *p* < 0.001 (one‐way ANOVA with post hoc Tukey test). D) qRT‐PCR analysis showing the mRNA levels of *Il1b* and *Nlrp3*. *n* = 6 per group. *ns*, not significant; ^***^, *p* < 0.001 (one‐way ANOVA with post hoc Tukey test). E) Immunoblotting analysis showing the protein levels of NLRP3, cleaved Caspase‐1, and cleaved IL‐1β in BMDM. *n* = 3 per group. *ns*, not significant; ^**^, *p* < 0.01; ^***^, *p* < 0.001 (one‐way ANOVA with post hoc Tukey test). F) Immunoblotting analysis showing the protein levels of cleaved Caspase‐1 and cleaved IL‐1β in heart tissues. *n* = 3 per group. ^*^, *p* < 0.05; ^**^, *p* < 0.01 (one‐way ANOVA with post hoc Tukey test). GABA, γ‐aminobutyric acid; LPS, Lipopolysaccharide; *Nos2*, Inducible nitric oxide synthase; *Tnf*/TNF‐α, Tumor necrosis factor‐α; *Nlrp3*/NLRP3, NOD‐, LRR‐ and pyrin domain‐containing protein 3; *Il1b/*IL‐1β, Interleukin‐1β; Caspase‐1, cysteine aspartate specific protease‐1; HSP90, heat shock protein 90.

We postulated that the reduced number of proinflammatory M1 macrophages by GABA gavage may be a result of GABA's inhibition of macrophage polarization in vivo. Accordingly, we further carried out an in vitro experiment using cultured bone marrow‐derived macrophages (BMDM) representing circulatory macrophages to test whether GABA might inhibit macrophage polarization toward the proinflammatory M1 phenotype. BMDM were treated with lipopolysaccharide (LPS) to induce macrophage polarization toward the M1 phenotype in the presence or absence of GABA. We found that the proportion of macrophages that polarized to proinflammatory M1 phenotype stimulated by LPS was decreased upon the addition of GABA, as indicated by decreased *Nos2* and *Tnf* mRNA levels (**Figure** **6**A). Compared with the vehicle group, LPS stimulation resulted in significantly increased mRNA levels of an M1 marker, nitric oxide synthase‐2 (*Nos2*) (*p* < 0.001), and an M1‐related proinflammatory cytokine, tumor necrosis factor (*Tnf*) (*p* < 0.001), both of which were significantly reversed by GABA treatment in BMDM (*p* < 0.001 and *p* < 0.001, respectively). This reduction of M1 macrophage polarization was further reflected by the changes in the expression levels of TNF‐α in BMDM by flow cytometry analysis, as the increased TNF‐α levels stimulated by LPS was reversed upon the addition of GABA (*p* < 0.001, Figure 6B).

As the activation of NLRP3 inflammasome is an important component of the innate immune system responsible for the activation of inflammatory responses and capable of inducing macrophage polarization toward M1,^[^ ^27^, ^28^ ^]^ we next examined the release of IL‐1β of BMDM by ELISA in the cell culture supernatants. We found that IL‐1β secretion was significantly increased by LPS stimulation compared to the vehicle group (*p* < 0.001) and this LPS‐stimulated increase of IL‐1β secretion was suppressed upon GABA treatment (*p* < 0.001) (Figure 6C), suggesting that GABA suppressed the activation of NLRP3 inflammasome induced by LPS. NLRP3 inflammasome activation requires both priming and activation signals.^[^ ^29^, ^30^ ^]^ Accordingly, we found that GABA treatment did not significantly reverse LPS‐stimulated *Nlrp3* and *Il1b* gene upregulation (*p* = 0.4869 and *p* = 0.7724, respectively) (Figure 6D), suggesting that GABA was not involved in the NLRP3 inflammasome priming stage. Subsequently, we examined NLRP3 inflammasome components in BMDM stimulated by LPS in the presence or absence of GABA by western blotting. We found that GABA treatment significantly reversed the LPS‐induced increase of cleaved Caspase 1 (*p* < 0.01) and IL‐1β (*p* < 0.01) at the protein level (Figure 6E), but not NLRP3 (*p* = 0.1143) (Figure 6E), indicating that GABA plays a vital inhibitory role in the NLRP3 inflammasome activation stage in LPS‐stimulated BMDM. To evaluate whether NLRP3 inflammasome activation could also be inhibited by GABA in mouse hearts, we measured the protein levels of cleaved Caspase 1 and IL‐1β in heart tissues. We found that both of them increased after I/R (*p* < 0.05 and *p* < 0.05, respectively), and were suppressed by GABA gavage (*p* < 0.01 and *p* < 0.05, respectively) (Figure 6F). Taken together, these data suggest that GABA inhibits the polarization of macrophages toward the proinflammatory M1 phenotype by suppressing the activation of NLRP3 inflammasome.

### 2.5 GABA Suppressed NLRP3 Inflammasome Activation by Inhibiting Macrophage Lysosomal Leakage

**Figure 7:**
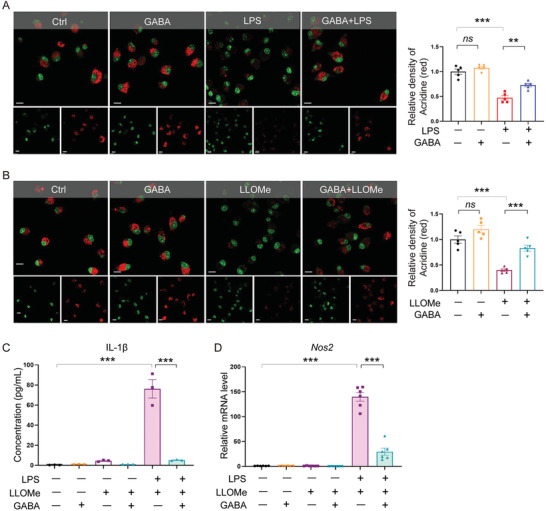
GABA suppressed NLRP3 inflammasome activation by inhibiting macrophage lysosomal leakage. A) Acridine orange staining of BMDM under LPS stress showing lysosomal leakage. As a cell‐permeable green fluorophore that can be protonated and trapped in acidic vesicular organelles, acridine orange fluoresces red in intact lysosomes and fluoresces green elsewhere that is less acidic. Quantitative data shown as the mean ± SEMs to the right. *n* = 5 per group. *ns*, not significant; ^**^, *p* < 0.01; ^***^, *p* < 0.001 (one‐way ANOVA with post hoc Tukey test). Scale bar, 10 µm. B) Acridine orange staining of BMDM treated with LLOMe or PBS in the presence or absence of GABA. Red fluorescence indicates intact lysosomes. Quantitative data are shown as the mean ± SEMs to the right. *n* = 5 per group. *ns*, not significant; ^***^, *p* < 0.001 (one‐way ANOVA with post hoc Tukey test). Scale bar, 10 µm. C) Measurement of IL‐1β secretion in cell culture supernatants by ELISA. Data are shown as the mean ± SEMs, *n* = 3 per group. ^***^, *p* < 0.001 (one‐way ANOVA with post hoc Tukey test). D) qRT‐PCR analysis of the mRNA levels of *Nos2*. *n* = 6 per group. ^***^, *p* < 0.001 (one‐way ANOVA with post hoc Tukey test). GABA, γ‐aminobutyric acid; LPS, Lipopolysaccharide; *Nos2*, Inducible nitric oxide synthase; IL‐1β, Interleukin‐1β.

Lysosomal leakage has often been implicated in and recognized as a driver of NLRP3 inflammasome activation responsible for acute and chronic inflammation in many common diseases.^[^ ^31^, ^32^, ^33^ ^]^ Accordingly, we hypothesized that GABA suppressed the NLRP3 inflammasome activation in macrophages by inhibiting lysosomal leakage. To test this hypothesis, we evaluated lysosomal leakage by staining BMDM with acridine orange, we found that LPS stimulation induced lysosomal leakage in BMDM, which was attenuated by GABA treatment (*p* < 0.01 and *p* < 0.05, respectively) (**Figure** **7**A; Figure S4A,B, Supporting Information). Considering that LPS may induce lysosomal leakage through indirect mechanisms, we further used L‐leucyl‐L‐leucine methyl ester (LLOMe), a lysosomotropic reagent that polymerizes inside lysosomes to induce lysosomal leakage,^[^ ^34^, ^35^ ^]^ to investigate the inhibitory effect of GABA on lysosomal leakage by staining BMDM with acridine orange. We found that LLOMe‐induced lysosomal leakage in BMDM was also attenuated by GABA treatment (*p* < 0.001) (Figure 7B). These data suggest that GABA inhibits lysosomal leakage in macrophages.

Lastly, to evaluate whether the inhibition of lysosomal leakage is vital for GABA's suppression of NLRP3 inflammasome activation and M1 macrophage polarization, we examined the release of IL‐1β of BMDM treated with LPS and LLOMe in the presence or absence of GABA by ELISA in the cell culture supernatants. We found that IL‐1β was significantly increased in response to LPS and LLOMe stimulation, compared to the vehicle group (*p* < 0.001) and this increase was suppressed upon GABA treatment (*p* < 0.001) (Figure 7C). In addition, the mRNA expression level of an M1 marker *Nos2* was upregulated when treated with LPS and LLOMe (*p* < 0.001) (Figure 7D), and this upregulation was reversed upon GABA treatment (*p* < 0.001) (Figure 7D), indicating that GABA's inhibition of lysosomal leakage is seminal for its suppression of NLRP3 inflammasome activation and M1 macrophage polarization. Taken together, these data suggest that GABA inhibits lysosomal leakage in macrophages and thus suppresses NLRP3 inflammasome activation and proinflammatory M1 macrophage polarization.

## 3 Discussion

Here, aiming to confer protection against acute coronary heart disease, we found that prophylactic treatment of the mouse model of I/R with *L. reuteri*, a common probiotic species that is easy to obtain and maintain, has significant cardiac protective effects against I/R, an important complication after MI. Prophylactic treatment with *L. reuteri* led to significantly reduced cardiac injury in terms of reduction in cardiac functions and importantly reduced the level of inflammation after I/R. We further found that GABA, one of the most‐studied metabolites from *L. reuteri* and other probiotic bacteria, was elevated in the serum of the mice gavaged with *L. reuteri*, and that the protective effect of this bacteria after I/R was mediated through GABA. Our study demonstrated the effect of GABA in reducing key indices for heart injuries and inflammation after I/R; we also showed that GABA conditioned the polarization of macrophages and reduced the number of proinflammatory M1 cells, which was indispensable for its cardioprotective effect. Within macrophages, we found that GABA suppressed NLRP3 inflammasome activation and the differentiation toward a proinflammatory status by inhibiting lysosomal leakage.

The inflammatory response after myocardial ischemia‐reperfusion injury is necessary for cardiac repair, but excessive inflammation can participate in pathophysiological processes such as cardiac remodeling and heart failure after myocardial infarction.^[^ ^20^, ^21^ ^]^ Here, the effect of GABA in reducing heart inflammation after I/R by macrophage modulation is promising and expands our current knowledge of this important molecule. Long recognized as a neurotransmitter that suppresses neural activity, GABA is increasingly being recognized for its pleiotropic effects on metabolism and immune functions.^[^ ^36^, ^37^, ^38^, ^39^ ^]^ For instance, in type 1 and type 2 diabetes, GABA has shown the potential to stimulate insulin secretion in beta‐cells, via activation of GABA_A_ and GABA_B_ receptors.^[^ ^40^, ^41^ ^]^ More recently, it has been found that B cells produce GABA and consequently suppress cytotoxic T cells and macrophages in cancer, thus inhibiting anti‐cancer immunity.^[^ ^39^ ^]^ Our study demonstrated the effect of GABA in shaping the polarization of macrophages and reducing the number of proinflammatory M1 cells, thus conferring its cardioprotective effect. Based on these findings of expanded functions of GABA, we propose that GABA can be applied for the prevention and/or treatment of diseases in which NLRP3‐centered inflammation and polarization toward M1 macrophages are to be avoided or reduced.

Among the many inflammatory mediators that are implicated in the early stage of I/R, IL‐1β is one of the early and most prominent mediators and the inhibition of IL‐1β signaling protects against MI injury.^[^ ^20^, ^42^, ^43^ ^]^ Consistent with the notion that IL‐1b is the main downstream molecule triggered by NLRP3 inflammasome,^[^ ^44^, ^45^, ^46^ ^]^ our study revealed that GABA not only reduced the levels of inflammatory cytokines in I/R hearts and M1‐polarized macrophages, but also suppressed macrophage NLRP3 inflammasome activation and IL‐1b release. While the roles of lysosomal leakage in response to pathogens and NLRP3 inflammasome activation have been widely recognized, our study also showed that the perseveration of lysosomes against leakage by GABA in macrophages contributes to the suppression of NLRP3 inflammasome activation. Whether GABA's effect on lysosomal leakage is cell type‐specific (i.e., only in macrophages or in more cell types) and context‐dependent (i.e., only under inflammation) requires continual study.

## 4 Conclusion

To summarize, our study demonstrates that a *L. reuteri* pretreatment attenuates acute ischemic cardiac injury, and identifies GABA as a cardioprotective metabolite that confers anti‐inflammatory effects by affecting lysosomal leakage, NLRP3 inflammasome activation, and macrophage polarization. Our study paves the way for the application of *L. reuteri* and other probiotics, and GABA directly, in reducing acute ischemic cardiac injury and possibly other complications of cardiovascular diseases. Considering the high diversity of gut microbial species and the large reservoir of metabolites from gut microbiome, it is worthy to investigate additional strains with protective effects or even therapeutic impacts and provide important therapeutical options for clinical usage.

## 5 Experimental Section

### Animals

All the C57BL/6J mice used in this study were obtained from SPF (Beijing) Biotechnology Co., Ltd. (Beijing, China). All the animal protocols were approved by the Institutional Care and Ethical Committee of the Institute of Zoology, Chinese Academy of Sciences. Eight‐ to ten‐week‐old male C57BL/6J mice under different treatments were housed in ventilated cages and fed an autoclaved diet under a strict 12‐h light cycle in an SPF animal facility. Mice were euthanized by isoflurane inhalation followed by cervical dislocation.

### Bacterial Strains and Oral Administration

A combination of antibiotics including vancomycin (0.125 mg mL^−1^ day^−1^) (1404‐94‐9, Sigma–Aldrich, USA), neomycin (0.25 mg mL^−1^ day^−1^) (1405‐10‐3, Macklin, China), metronidazole (0.25 mg mL^−1^ day^−1^) (443‐48‐1, Solarbio, China), and ampicillin (0.25 mg mL^−1^ day^−1^) (7177‐48‐2, Solarbio, China) were mixed in drinking water and given to mice ad libitum for 7 days to deplete their gastrointestinal microbiota.^[^ ^47^ ^]^ Aspartame (0.1 mg mL^−1^ day^−1^) (A801109, Macklin, China) was added to make the mixture more palatable. Mice were kept in a completely bacteria‐free environment following antibiotic cocktail treatment.

Experimental *Lactobacillus reuteri* strains (CICC 6126) used in this study were acquired from the China Center of Industrial Culture Collection (CICC). The stock culture was maintained at −80 °C in 20% glycerol before use. The bacteria were propagated twice in *Lactobacilli* MRS broth (Hopebio, China) overnight at 37 °C before the experimental procedures. Cell pellets were obtained by centrifuging at 8000 *g* for 10 min at 4 °C and resuspended in oxygen‐free PBS with a final density of 1 × 10^9^ CFU per mL. Mice were daily gavaged with 200 µL suspension solution for the duration indicated in the study.

### Quantification of 16S rDNA

The evaluation of antibiotic depletion was conducted using total 16s rDNA. DNA extraction from the fecal samples was performed with a DNeasy PowerSoil Pro Kit (47 016, QIAGEN, Germany) following the manufacturer's instructions. The bacterial 16S rRNA genes was then amplified (forward primer 5'‐TCCTACGGGAGGCAGCAGT‐3' and reverse primer 5'‐GGACTACCAGGGTATCTAATCCTGTT‐3') and *Lactobacillus reuteri* was quantified with species‐specific primers (forward primer 5′‐CAGACAATCTTTGATTGTTTAG‐3′; reverse primer 5′‐GCTTGTTGGTTTGGGCTCTTC‐3′). Quantitative real‐time PCR was performed on a 7500 Fast Real‐Time PCR System using the KAPA SYBR FAST Kit (KK4601, KAPA, USA). The amount of amplified DNA was determined using a 16S rDNA standard curve obtained by real‐time PCR from a serial dilution of *E. coli* DNA.

### Ischemia/Reperfusion Studies In Vivo and Echocardiography

The mice were anesthetized with 2% isoflurane. The chest cavity of the mice was opened at the fourth intercostal space and the pericardium was excised. Subsequently, a silk ligature was positioned distal to the left atrial appendage, spanning the sternal portion of the left ventricle, which included the left anterior descending coronary artery for 40 min. The suture was then released to allow for reperfusion of the ischemic area. Following the surgical procedure, the chest was closed and the mice were administered lidocaine (1.5 mg kg^−1^, s.c.) for post‐operative analgesia. After recovering, the animals were returned to their feeding cages.

A Visual Sonics Vevo 3100 imaging system (Visual Sonics, Inc.) equipped with a 30‐MHz transducer (MX400) was utilized to conduct transthoracic echocardiography in order to assess mouse cardiac function 1 week following ischemia/reperfusion (I/R). Mice were all anesthetized with 1.5% isoflurane in 100% O_2_ gas. 2D‐targeted M‐mode traces were acquired at the papillary muscle level to measure left ventricular ejection fraction (LVEF), left ventricular fractional shortening (LVFS), left ventricular end‐diastolic dimension (LVEDD), and left ventricular end‐systolic dimension (LVESD) using cardiac echocardiography software. The measurements were calculated using Image J (Image J2, Fiji) from three distinct cardiac cycles. Mice were euthanized via cervical dislocation at the end of the experiments and their organs were removed, rinsed in heparinized saline, dried, and weighed.

### Electrocardiogram (ECG) Analysis

ST‐segment elevation was measured by a surface ECG using a single‐channel, calibrated (1 mV reference) amplifier (KLS‐1, Kardiotek Jacketed Telemetry System, Shanghai Kexin Medical Biotechnology). To maintain consistency in ECG amplitude, the animal's position was kept unchanged during ECG registration. ECG alterations, especially ST‐segment elevation or depression, were documented from 30 consecutive cardiac cycles at both baseline and three days after I/R, with premature beats or instances of ventricular tachycardia/fibrillation excluded from the quantitative analysis.

### Histological Analysis of Mouse Heart

Heart tissues were freshly fixed in 4% paraformaldehyde (PFA) (DF0131, Leagene Biotechnology), dehydrated through graded alcohols, embedded in paraffin wax, and sectioned at 5 µm. The sections were then deparaffinized using xylene and rehydrated through a sequential ethanol process. Cardiac fibrosis was evaluated by picrosirius red staining and Masson trichrome's staining. For picrosirius red staining, rehydrated sections were incubated in picrosirius red dye (DC0041, Leagene Biotechnology, China) for 60 min at room temperature followed by a Weigert iron hematoxylin solution (ZLI‐9610, ZSGB‐BIO, China) for 10 min at room temperature. The sections were washed with running water, dehydrated through an ethanol serial and xylene, and mounted using a neutral balsam mounting medium (N116470, Aladdin, China). For Masson trichrome's staining, the rehydrated sections were immersed in a Weigert iron hematoxylin solution for 10 min at room temperature and washed with running water. Subsequently, the sections were incubated in Biebrich Scarlet‐Acid Fuchsin (TRM‐1, Trichrome Stain Kit, Scy Tek, USA) for 15 min at room temperature and briefly rinsed in deionized water. The sections were then incubated in a phosphotungstic‐phosphomolybdic acid solution (1:1) (TRM‐1, Trichrome Stain Kit, Scy Tek, USA) for 5 min at room temperature. The sections were then immersed in an aniline blue solution (TRM‐1, Trichrome Stain Kit, Scy Tek, USA) for 10 min at room temperature and 1% acetic acid (TRM‐1, Trichrome Stain Kit, Scy Tek, USA) for 1–2 min at room temperature before dehydrated through an ethanol serial and xylene and mounted using a neutral balsam mounting medium (N116470, Aladdin, China). The stained sections were captured using a Zeiss Axio Observer Z1 widefield microscope. The quantification of the fibrotic area in the left ventricle was determined by analyzing the blue‐colored regions with the assistance of Image J (Image J2, Fiji).

For immunohistological staining, the rehydrated sections were incubated with 3% H_2_O_2_ for 10 min at room temperature. Antigen retrieval was then carried out using a microwave and sodium citrate buffer. Subsequently, the sections were allowed to cool to room temperature and were incubated with a blocking buffer (5% bovine serum, A8020, Solarbio, China) for 1 h at room temperature. This was followed by overnight incubation at 4 °C with primary antibodies against TNF‐α (1:200, 17590‐1‐AP, Proteintech, China) or IL‐1β (1:200, 16806‐1‐AP, Proteintech, China). The following day, the sections were washed three times in PBS for 10 min each time and incubated with a biotinylated goat anti‐rabbit secondary antibody (SP‐0022 SP Kit, Bioss, China) for 1 h at room temperature. The sections were then washed three times in PBS for 10 min each time and incubated with streptavidin horseradish peroxidase (HRP) (SP‐0022 SP Kit, Bioss, China) for 30 min at room temperature before the visualization using 3,3′‐diaminobenzidine tetrahydrochloride (DAB) (8059S, Cell Signaling, USA).

Prior to dehydration through an ethanol serial, the sections were counterstained with hematoxylin and mounted using a neutral balsam mounting medium (N116470, Aladdin, China). Negative controls were generated by using IgG instead of the forementioned primary antibodies. The stained sections were captured using an Olympus microscope IX73, and the quantification of TNF‐α and IL‐1β signals was performed using Image J (Image J2, Fiji).

### Measurement of cTnT, GABA, and Cytokine Levels by ELISA

For measurement in the serum, blood was withdrawn from the mouse facial vein at indicated time points in the study, left undisturbed at 4 °C for 4 h to clot, and centrifuged at 1000 *g* for 10 min. The resultant supernatant was collected for subsequent analysis. For the measurement in tissues, heart tissues were homogenized in pre‐cooled PBS and centrifuged at 5000 *g* for 10 min and the resultant supernatant was collected for downstream analyses. In the case of BMDM, the cell supernatants were collected for downstream analyses. The GABA level was measured by using an ELISA kit (RK09121, Abclonal, China) following the manufacturer's instructions. The levels of cTnT (F7649B, Yutong, China), IL‐1β (RK00006, Abclonal, China), TNF‐α (RK00027, Abclonal, China), IL‐6 (RK00008, Abclonal, China), and IFN‐γ (RK00019, Abclonal, China) were detected by using ELISA kits following the manufacturer's instructions.

### Flow Cytometry Analysis of Immune Cells in the Heart

Single‐cell suspension of the mouse heart was prepared as previously described.^[^ ^48^ ^]^ The mouse heart was initially injected with EDTA buffer (130 mm NaCl, 5 mm KCl, 0.5 mm NaH_2_PO_4_, 10 mm HEPES, 10 mm Glucose, 10 mm BDM, 10 mm Taurine, 5 mm EDTA), with the inferior vena cava open in the cavity. Subsequently, the aorta was clamped, and the heart was isolated and perfused with EDTA buffer, perfusion buffer (130 mm NaCl, 5 mm KCl, 0.5 mm NaH_2_PO_4_, 10 mm HEPES, 10 mm Glucose, 10 mm BDM, 10 mm Taurine, 1 mm MgCl_2_), and collagenase buffer (0.5 mg mL^−1^ Collagenase 2, LS004176, Worthington, USA; 0.5 mg mL^−1^ Collagenase 4, LS004188, Worthington, USA). After removing the clamp, heart tissues were gently pulled into 1 mm^3^ pieces using forceps and dissociated by gentle pipetting in a dish with the forementioned collagenase buffer. Following 1 h of digestion, enzymes were deactivated by the addition of stop buffer (perfusion buffer with the addition of sterile fetal bovine serum (FBS)). The resulting cell suspensions were filtered through a 100 µm cell strainer. Cardiomyocytes were gravity‐settled for 20 min and the supernatant containing other types of cells was then collected and centrifuged at 300 *g* for 5 min at 4 °C. The resultant supernatant was discarded, and the cell pellet was resuspended for downstream analysis. Cells were stained with monoclonal antibodies for 30 min at room temperature and after being treated with Fcg‐blocking antibody anti‐mouse CD16/32 (Biolegend, CA, USA) for 10 min at 4 °C. The CD45^+^ gate was used for all immune cells. Neutrophils were gated as CD11b^+^Ly6G^+^. Macrophages were gated as CD11b^+^F4/80^+^ and divided by CD11b^+^F4/80^+^Ly6C^high^ and CD11b^+^F4/80^+^Ly6C^low^. B cells and T cells were gated by CD11b^−^B220^+^ and CD11b^−^TCR‐β^+^, respectively. All samples were analyzed on a FACS Canto II Cell analyzer (BD Biosciences, NJ, USA). Analysis of acquired data was performed with the FlowJo software (FlowJo 10.8.1).

The antibodies used were: BV421 anti‐mouse CD45 1:50 (103 134, Biolegend, USA), BV510 anti‐mouse CD11b 1:200 (101 263, Biolegend, CA, USA), APC anti‐mouse B220 1:50 (103 212, Biolegend, CA, USA), PE/Cy7 anti‐mouse TCR‐β (109 222, Biolegend, CA, USA), BV605 anti‐mouse Ly6C 1:50 (128 036, Biolegend, CA, USA), FITC anti‐mouse Ly6G 1:50 (127 605, Biolegend, CA, USA), PE anti‐mouse F4/80 1:50 (123 109, Biolegend, CA, USA).

### Macrophage Depletion with Clodronate Liposomes

Mice were intravenously injected with 150 µL clodronate liposomes (CP‐005‐005, Liposoma, The Netherlands) at 2 days before I/R surgery to deplete macrophages as previously described.^[^ ^25^, ^26^ ^]^

### BMDM Isolation and Polarization

Murine bone marrow‐derived macrophages (BMDM) were isolated and cultured as previously reported.^[^ ^49^ ^]^ Bone marrow was obtained by flushing DMEM media through the femur, tibia, and hip bones of mice with a 25‐gauge needle. The bone marrow was then resuspended in red cell lysis buffer (NH4CL2009, TBD Science, China) for 3 min before being centrifuged, resuspended, and passed through a 70 µm cell strainer. Cells were plated at 1 × 10^6^ cells mL^−1^ in DMEM supplemented with 10% FBS, and 1% penicillin/streptomycin solution containing M‐CSF (50 ng mL^−1^, 78 057, STEMCELL Technologies) and maintained at 37 °C with 5% CO_2_ for 6 days, to allow the differentiation into macrophages. On day 7, cells were stimulated by LPS (Sigma–Aldrich, USA) at a concentration of 200 ng mL^−1^ for 3–6 h as appropriate to induce polarization into M1 macrophages. Subsequently, the cells were collected for downstream analyses. In the groups treated with GABA, the medium was supplemented with 10 mm GABA (Solarbio, Beijing, China) starting from 2 h prior to LPS stimulation until the end of the experiment. To activate NLRP3 inflammasome in LPS‐stimulated BMDM, cells were treated for an additional 2 h with 15 µM nigericin sodium salt (Selleck Chemicals, Texas, USA) after the LPS stimulation. For the quantification of macrophage polarization to M1 phenotype, BMDM were stained with fluorescently labeled antibodies (PE/Cy7 anti‐mouse CD11b, 1:100, 101 208, Biolegend, USA, APC anti‐mouse F4/80, 1:100, 123 115, Biolegend, USA, and PE anti‐mouse TNF‐α, 1:50, 506 305, Biolegend, USA). Macrophages were gated by CD11b^+^/F4/80^+^ and the mean fluorescence intensity (MFI) of TNF‐α signals was measured by flow cytometry.

### Detection of Lysosomal Leakage in BMDM

BMDM were treated with LPS at 200 ng mL^−1^ for 3 h or LLOME at 500 nm for 30 min to induce lysosomal leakage in the presence or absence of GABA as appropriate. Lysosomal leakage was detected with acridine orange^[^ ^50^ ^]^ or LysoTracker red DND‐99 (L7528, Thermo Fisher Scientific, USA) that specifically stains acidic compartments within a cell. In brief, BMDM grown on coverslips were stained with 2 µg mL^−1^ acridine orange (A1301, Thermo Fisher Scientific, USA) for 20 min or 50 nm LysoTracker red for 30 min at 37 °C in a humidified 5% CO_2_/95% air incubator and washed in PBS before the visualization. Loss of acridine orange staining (Ex 488 nm/Em 650 nm) or LysoTracker red (Ex 577 nm/Em 590 nm) was used as an indicator for lysosomal leakage. The images were captured with Andor Dragonfly 505 confocal spinning disk system. Cell nuclei were stained with Hoechst 33 342 (Ex 405 nm, Em 460 nm) (C1022, Beyotime, China). Quantitative analysis was performed with Imaris Viewer x64 9.6.0 software.

### Western Blotting

Myocardial tissue homogenates were prepared by homogenizing fresh or liquid nitrogen snap‐frozen heart tissues in Tissue Extraction Reagent II (FNN0071, Thermo Fisher Scientific, USA) supplemented with a protease inhibitor (0 589 297 0001, Roche, Switzerland) and a phosphatase inhibitor (0 490 683 7001, Roche, Switzerland). The homogenates were obtained from the supernatant after the centrifugation at 3800 *g* at 4 °C for 10 min. To prepare cell lysates, BMDMs were pelleted and lysed in RIPA lysis buffer (P0013E, Beyotime, China) on ice with protease and phosphatase inhibitors according to the manufacturer's protocol. Subsequently, 30 µg of protein lysates were size‐separated by 10% SDS‐PAGE, transferred to a 0.22 µm PVDF membrane (BSP0161, Pall, USA), and blocked with 5% skim milk (CN311911100, Coolaber, China) for 1 h at room temperature. The membrane was then incubated overnight at 4 °C with the primary antibody, followed by incubation with the secondary antibody for 1 h at room temperature. Finally, the protein bands were visualized using ECL chemiluminescence reagent (180‐5001, Tanon, China).

### RNA Extraction and Quantitative RT‐PCR

Total RNA was extracted from mouse hearts or cells by using TRIzol Regent (15 596 018, Thermo Fisher Scientific, USA) and subjected to complementary cDNA synthesis by using RevertAid Master Mix (M1631, Thermo Fisher Scientific, USA), following the manufacturer's instructions. PCR amplification products were assessed using TB Green Premix Ex Taq (Tli RNaseH Plus) (RR420D, Takara, Japan). The mRNA levels of genes were normalized to those of *Gapdh*. The sequences of primers used for RT‐PCR are listed below.

*Nos2* Fw: 5′‐CTCACTGGGACAGCACAGAA‐3′;

*Nos2* Re: 5′‐GCTTGTCTCTGGGTCCTCTG‐3′;

*Tnf* Fw: 5′‐TATGGCTCAGGGTCCAACTC‐3′;

*Tnf* Re: 5′‐CTCCCTTTGCAGAACTCAGG‐3′;

*Nlrp3 Fw*: 5′‐TAGCTTCTGCCGTGGTCTCT‐3′;

*Nlrp3 Re*: 5′‐AGGAGATGTCGAAGCAGCAT‐3′;

*Gapdh* Fw: 5′‐CATCACTGCCACCCAGAAGACTG‐3′;

*Gapdh* Re: 5′‐ATGCCAGTGAGCTTCCCGTTCAG‐3′.

### RNA Sequencing Analysis

Infarcted heart tissues were homogenized in TRIzol (Thermo Fisher Scientific, USA) and total RNA was used as input material for the RNA sample preparations. Sequencing libraries were generated using the NEBNext Ultra RNA Library Prep Kit for Illumina (E7530L, NEB, USA) following manufacturer's recommendations, and index codes were added to attribute sequences to each sample. The qualified libraries were pooled and sequenced on Illumina platforms with PE150 strategy (Novogene Bioinformatics Technology, Beijing, China), according to effective library concentration and data amount required. The differential expression genes (DEGs) were analyzed with the R package DESeq2 (version 1.12.3) and identified with absolute fold change ≥ 1.5 and adjusted *p*‐value < 0.05 (Benjamini–Hochberg method).^[^ ^51^ ^]^ The full set of processed detected genes was first examined using principal component analysis (PCA). With variance stabilizing transformation of the read counts, PCA was performed using the prcomp function of the R Stats Package (version 3.6.2). DEGs were subjected to volcano plots, heatmaps, and Gene Ontology (GO) enrichment analyses. The volcano plot and heatmap were visualized with the R packages ggplot2 (version 3.4.2) and pheatmap (version 1.0.12). Gene Ontology (GO) enrichment was determined with clusterProfiler (version 3.18.1) using R. The results of GO enrichment were visualized with ComplexHeatmap (version 2.10.0).

### Statistical Analysis

All statistical analyses were performed using GraphPad Prism 8.4 software (Prism, San Diego, CA, USA). To compare the two groups, either a one‐tailed unpaired *t*‐test or a one‐tailed Mann–Whitney test was used as appropriate. For comparisons involving more than two groups, a one‐way analysis of variance (ANOVA) followed by a post hoc Tukey test or a Kruskal–Wallis test with a post hoc Dunnett's test was used as appropriate. Data are presented as the mean ± SEMs or as individual data points. ^*^ *p* < 0.05, ^**^ *p* < 0.01, and ^***^ *p* < 0.001 are considered statistically significant.

## Conflict of Interest

The authors declare no conflict of interest.

## Supporting information

Supporting Information

## Data Availability

Data will be publicly available upon acceptance.
