# Supplementary material for: Prophylactic Supplementation with Lactobacillus Reuteri or Its Metabolite GABA Protects Against Acute Ischemic Cardiac Injury
Source: Adv Sci (Weinh). 2024 Mar 15;11(18):2307233. doi: 10.1002/advs.202307233 (PMC11095141; doi:10.1002/advs.202307233)
Supplement: Supplementary file 1 — Supporting Information [file ADVS-11-2307233-s001.pdf]

ADVANCED SCIENCE
Open Access

Supporting Information

for *Adv. Sci.*, DOI 10.1002/advs.202307233

Prophylactic Supplementation with *Lactobacillus Reuteri* or Its Metabolite GABA Protects Against Acute Ischemic Cardiac Injury

Jiawan Wang, Hao Zhang, Hailong Yuan, Siqi Chen, Ying Yu, Xuan Zhang, Zeyu Gao, Heng Du, Weitao Li, Yaohui Wang, Pengyan Xia, Jun Wang and Moshi Song**

Supplemental Materials

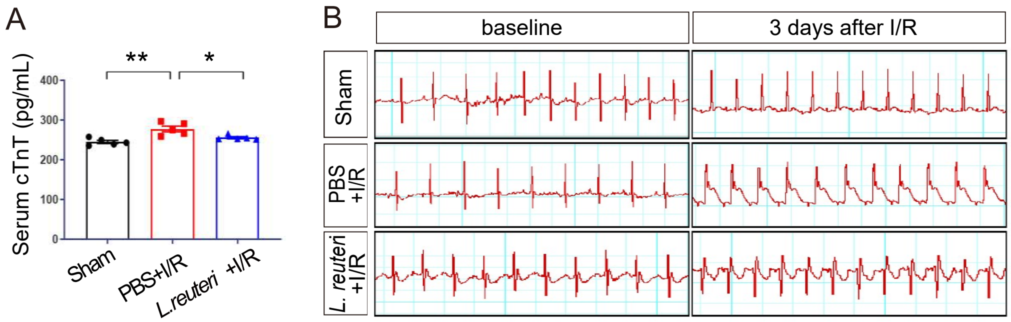

Figure S1. Pretreatment of *L. reuteri* alleviated cardiac damage after I/R.

(A) The left anterior descending artery ligation/reperfusion surgery was performed on mice from different groups to construct the myocardial I/R injury model. Serum cTnT levels measured by ELISA at seven days after I/R. n = 5 per group. *, $P<0.05$; **, $P<0.01$ (one-way ANOVA with post hoc Tukey test). (B) Electrocardiogram (ECG) traces (50 ms/div) were performed before and at three days after I/R in mice. ST-segment elevation lasted for at least three days after the surgery in I/R group, which returned to baseline at three days after the surgery in the group pretreated with *L. reuteri*. cTnT, cardiac troponin T; I/R, ischemia/reperfusion; *L. reuteri, Lactobacillus reuteri*.

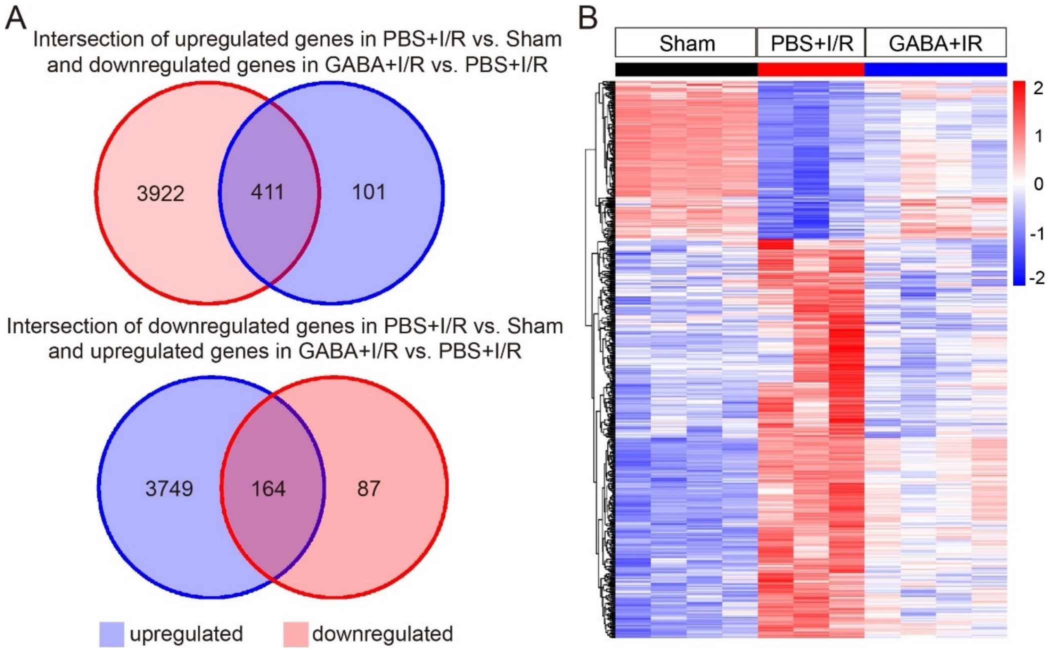

Figure S2. Transcriptional alterations by GABA treatment in mouse hearts after I/R.

(A) The upper Venn diagram showing the overlap between significantly upregulated genes in PBS+I/R versus Sham groups and significantly downregulated genes in GABA+I/R versus PBS+I/R groups. The lower Venn diagram showing the overlap between significantly downregulated genes in PBS+I/R versus Sham groups and significantly upregulated genes in GABA+I/R versus PBS+I/R groups. Differentially expressed genes were identified with an absolute fold change ≥ 1.5 and an adjusted p-value < 0.05. (B) Heatmap of the expression levels of genes differentially regulated in PBS+I/R versus Sham groups and rescued by GABA. Gene expression level was indicated by color ranging from blue (low expression) to red (high expression). GABA, γ-aminobutyric acid; I/R, ischemia/reperfusion; DEGs, differentially expressed genes.

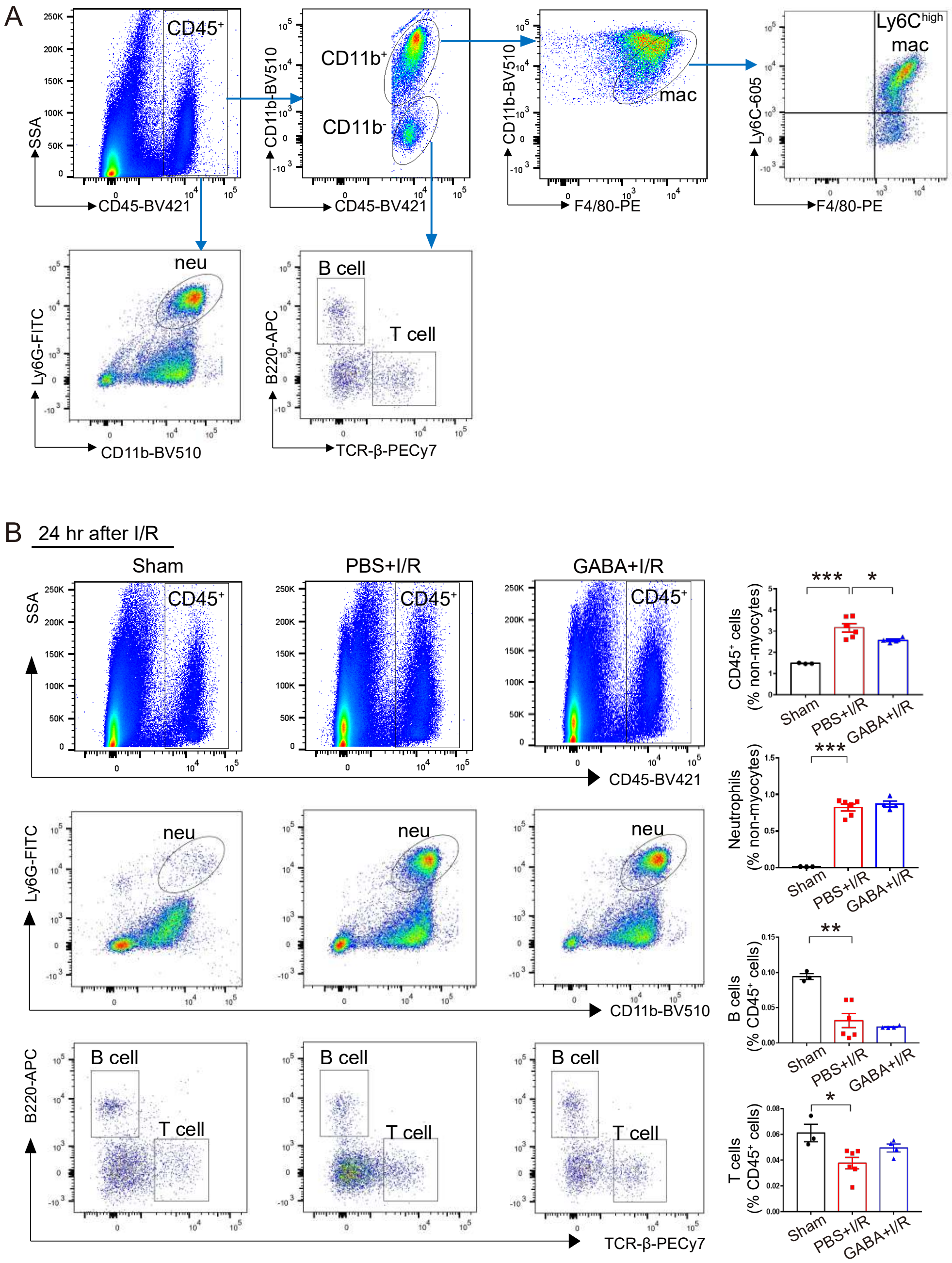

A
CD45+
CD11b+
CD11b-
mac
Ly6Chigh mac
SSA
CD45-BV421
CD11b-BV510
F4/80-PE
Ly6C-605
neu
B cell
T cell
Ly6G-FITC
B220-APC
CD11b-BV510
TCR-β-PECy7
B
24 hr after I/R
Sham
PBS+I/R
GABA+I/R
CD45+ cells (% non-myocytes)
Neutrophils (% non-myocytes)
B cells (% CD45+ cells)
T cells (% CD45+ cells)

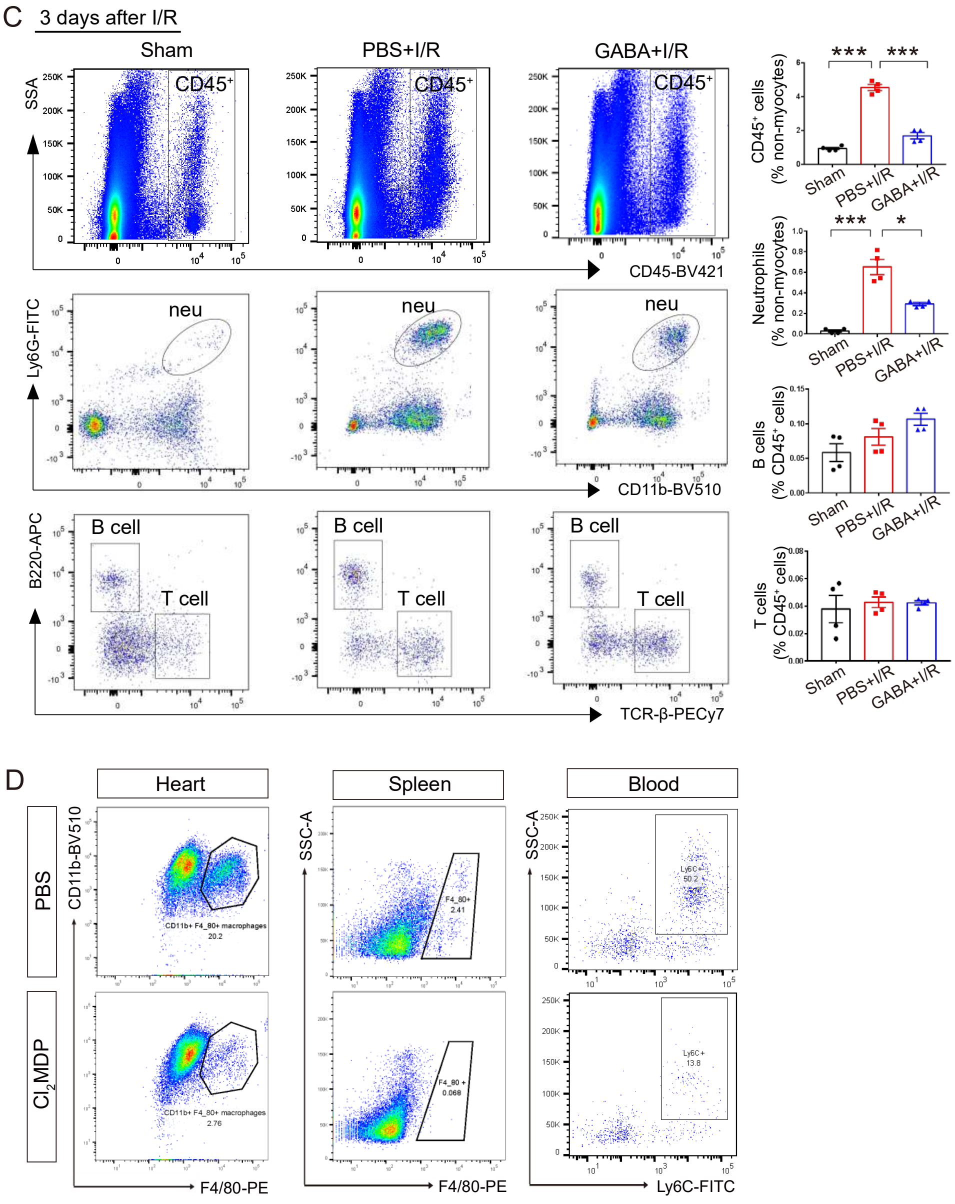

Figure S3. GABA mitigated I/R-induced inflammation mainly by affecting macrophages.

(A) Gating strategy identifying cardiac immune cells, including B cells, T cells, neutrophils, macrophages, and Ly6C^{high} macrophages. (B) Representative dot plots of CD45$^+$ immune cells, neutrophils, B cells and T cells in heart tissue at 24 hr after I/R (left panel). Quantification of the percentages of CD45$^+$ cells and neutrophils in all non-myocytes, B cells and T cells gated in CD45$^+$ cells (right panel, n ≥ 3). *, *P*<0.05; **, *P*<0.01; ***, *P*<0.001

(one-way ANOVA with post hoc Tukey test). (C) Representative dot plots of $CD45^+$ immune cells, neutrophils, B cells and T cells in heart tissue at three days after I/R (left panel). Quantification of the percentages of $CD45^+$ cells and neutrophils in all non-myocytes, B cells and T cells gated in $CD45^+$ cells (right panel, n > 3). *, *P*<0.05; ***, *P*<0.001 (one-way ANOVA with post hoc Tukey test). (D) Representative dot plots of circulating monocytes, $CD11b^+$ $F4/80^+$ macrophages in the spleen and heart before and after macrophage clearance with Cl_2MDP injection. GABA, γ-aminobutyric acid; mac, macrophage; neu, neutrophils; Cl_2MDP, clodronate liposome.

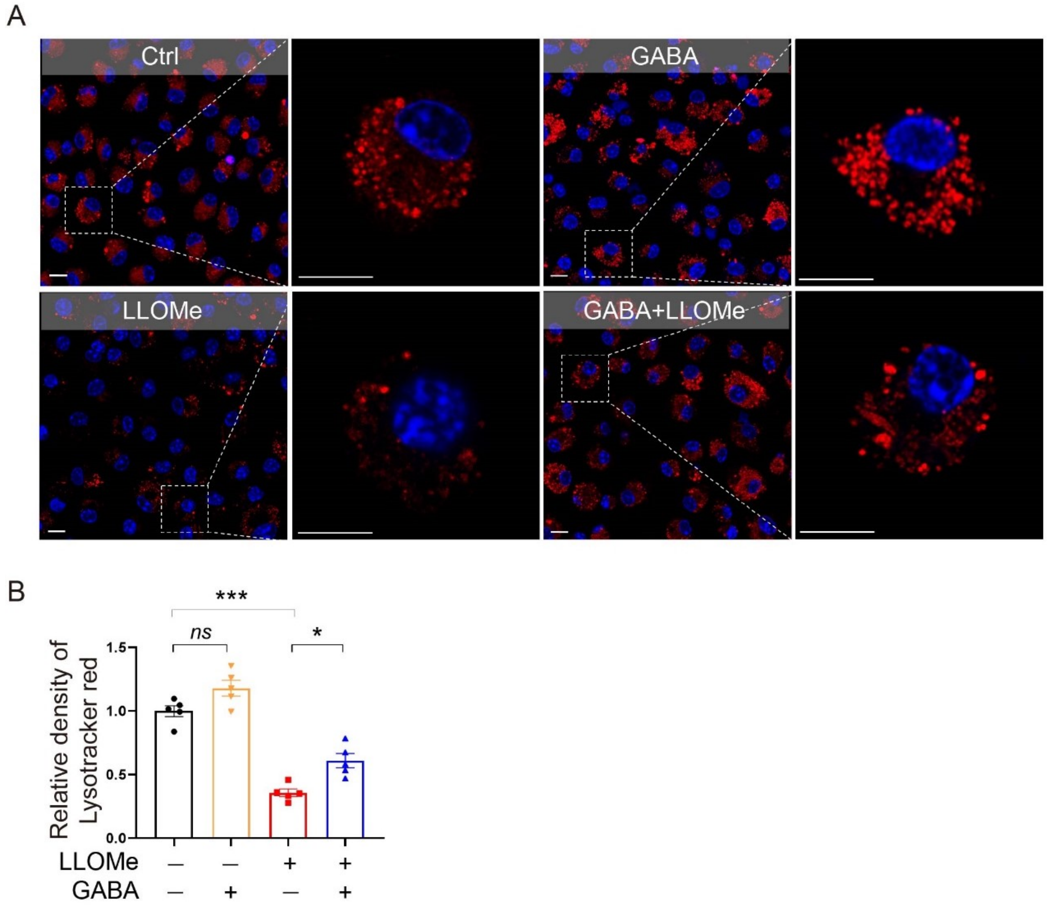

Figure S4. GABA inhibited macrophage lysosomal leakage.

(A) Representative images of BMDM stained with LysoTracker red in Ctrl, GABA, LLOMe, and GABA+LLOMe groups. Red fluorescence indicates intact lysosomes and the absence of red fluorescence indicates lysosomal leakage. Scale bar, 10 μm. (B) Quantification of relative density of LysoTracker red. Quantitative data shown as the mean ± SEMs to the right. n = 5 per group. *ns*, not significant; *, $P<0.05$; ***, $P<0.001$ (one-way ANOVA with post hoc Tukey test).
